# The Bacteriology of Common Colds

**Published:** 1909-12-11

**Authors:** 


### PATHOLOGY.

### THE BACTERIOLOGY OF COMMON COLDS.

The micro-organisms which naturally inhabit the nasal cavities and throats of healthy individuals are legion, and it is a problem of no small difficulty to determine which of the many bacteria present are to be considered pathogenic. Much evidence is accumulating with regard to four organisms in particular however—namely, micrococcus catarrhalis, micrococcus paratetragenus, bacillus septus (bacillus coryzæ segmentosus, Cautley), bacillus Friedländer, and probably two others, namely, bacillus influenzæ and the pneumococcus. The first-named, M. catarrhalis, is well established as a cause of common colds, and also of much more serious conditions, such as influenza, bronchitis, and pneumonia, sometimes causing cough so violent as to suggest a paroxysm of whooping-cough. It is a large coccus occurring in pairs. Its colonies are whitish-yellow and opaque, and attain a considerable size. It grows best on blood-agar, and usually fails to grow on gelatin. M. paratetragenus has been very frequently observed in epidemics during the last two winters; it is very common in pharyngeal cells, and masses of it can be seen in the sputum in many cases of phthisis and other pulmonary affections. It is a coccus of extremely variable size. Colonies may readily be obtained on ordinary agar in eight to twelve hours. They are softer and less adherent than colonies of M. catarrhalis. Of all the germs described as occurring in common colds, B. septus has least claim to be a cause of coryza; it causes rather a mild pharyngitis, with painful throat, muscular pains, and often severe nervous depression, but with no temperature and little or no nasal catarrh. It is probably a common cause of "stiff neck," "muscular rheumatism," and various transient muscular pains. It is a diphtheroid bacillus, found both in the nose and throat of patients suffering from pharyngitis, but its most common situation is in the flat cells of the pharyngeal epithelium. B. Friedländer occurs in many acute and chronic colds, and is said to be the cause of very profuse coryza. Opsonic work appears to prove that the B. septus, M. catarrhalis, and B. Friedländer are all pathogenic in certain cases of common colds, and it is certain that in the majority of cases that patients infected with these germs can be successfully treated by vaccine therapy.

